# The perils of boarding: A call to achieve parity in the delivery of acute psychiatric services for children with COVID-19

**DOI:** 10.34172/hpp.2023.04

**Published:** 2023-04-30

**Authors:** Aysha Jawed, Rachel Boro-Hernandez

**Affiliations:** Johns Hopkins Children’s Center, Department of Pediatric Social Work, Baltimore, MD, USA

**Keywords:** Acute crisis stabilization, Psychiatry, COVID-19, Pediatric, Inpatient, Boarding crisis

## Abstract

Boarding across pediatric healthcare systems is on the rise during the pandemic. Children with positive COVID-19 test results awaiting psychiatric placements in the emergency department or medical unit settings are at increased risk for decompensation with unmet psychiatric needs during a time of crisis marked by vulnerability. There is scant literature unveiling best practices on delivery of care for these patients to achieve acute crisis stabilization. Recent studies have uncovered substantial increases in mental health disorders among children during the pandemic compared to previous incidence and prevalence rates prior to the pandemic. From the published literature, two healthcare systems have initiated long-term planning, development, and implementation of biodome psychiatric units for patients with COVID-19 in need of acute crisis stabilization services. We sampled 100 acute inpatient child and adolescent psychiatric programs to discern their post-COVID positive clearance policies for admission. Findings were mixed among days of quarantine required, symptomology, covid-designated spaces vs. self-isolated rooms for psychiatric treatment, number of COVID negative retests, and additional considerations. We also review a range of considerations and recommendations for clinical practice and the health system in achieving parity in mental health care for these patients which in turn could contribute towards mitigating the rising global mental health crisis. Furthermore, increasing access to acute psychiatric services for these patients will also contribute towards the larger goal of the World Health Organization, Sustainable Developmental Goals of the United Nations, and Healthy People 2030 in increasing accessibility, quality and equity of mental health care for individuals on both global and national frontiers.

## Introduction

 Across pediatric healthcare systems, boarding in the hospital across emergency departments and inpatient medical units has grown tremendously during the existing COVID-19 pandemic.^1,2^ Children are boarding for a wide range of reasons inclusive of awaiting psychiatric, out-of-home, rehabilitation, and eating disorder inpatient placements. Unfortunately in the context of our pandemic era, we are observing a significant increase in children awaiting placement across acute healthcare systems given their positive COVID-19 status at time of presentation to the emergency department or during admission. The COVID-19 clearance policy based on governmental guidelines that continues to predominate across the healthcare landscape for transition to inpatient child psychiatric placement is ten days following time of positive test result. This circumstance is creating a sequalae of issues for our healthcare system and moreover for our patients who are not able to receive timely and needed treatment. Furthermore for patients awaiting psychiatric placement, this boarding crisis secondary to COVID positivity rates is contributing to the growing unprecedented mental health crisis worldwide. It follows that in many ways, we are observing a crisis within a crisis – an increased mental health crisis from systemic limitations in access to care amidst the global pandemic crisis.

## Rising Mental Health Crisis estimates

 One study that examined anxiety and depression rates among children between 5 to 17 years of age both before and during the pandemic, findings revealed that the rates had increased to nearly 1 in 6 children suffering from either or both anxiety and depression which marked an increase in these psychiatric morbidities by a little over 2% than pre-pandemic times.^3^ Another study uncovered an increase by almost 7% among children presenting to the emergency department with suicide attempt or self-injury among a sample of nearly 8000 children during the pandemic.^4^ In the same study, there was also a nearly 2% increase in visits to the emergency department among children presenting with symptomology consistent with disruptive, impulse control, and conduct disorders during the pandemic than before. Of note, several of these children necessitated a higher level of psychiatric care warranting inpatient placement. In a different study that compared rates of eating disorders among children both before and during the pandemic, trends revealed that there was a substantial uptake in the incidence of eating disorders among adolescents by nearly 7% for the first almost 18 months of the pandemic prior to a decline in prevalence thereafter, although still higher than pre-pandemic prevalence rates.^5^ The implications of these findings reveal that the pandemic has contributed to exacerbating the existing mental health crisis in the pediatric population.

## Organizational guidelines for inpatient psychiatric care of COVID-19 positive patients

 The Substance Abuse and Mental Health Services Administration’s (SAMSHA) guidelines present recommendations to create both COVID-19 and non-COVID-19 spaces across inpatient healthcare settings as the basis to increase access to acute crisis stabilization services for patients who need them the most.^6^ These guidelines also recognize that inpatient psychiatric care can be inevitable for patients and suggests measures to protect patients, their families, and staff amidst positive COVID-19 test results. The existing published literature yields one hospital in the United States that has successfully implemented these recommendations into the delivery of inpatient psychiatric services. The Rochester University Medical Center created a standalone inpatient psychiatric unit specifically for patients with COVID-19 influenced by an inpatient psychiatric unit of this nature in Israel.^7,8^ The layout of the units in both of these hospitals follows the biodome model that has already existed and continues to exist across healthcare systems in the United States and across the world. The Centers for Disease Control and Prevention (CDC) also presents guidelines that continue to be ever-changing with respect to number of days for quarantine following a COVID-positive test result based on vaccination status as well as additional behavioral recommendations (e.g. social distancing and masking parameters).^9^ At this point in time, the CDC recommends 5 days of quarantine following a positive COVID-19 test result.^9^

## Findings from a sample of acute inpatient child and adolescent psychiatric programs

 As part of determining variations in post-COVID-19 clearance policies across acute inpatient child and adolescent psychiatric programs, we contacted 100 programs across the USA in February 2023 to determine their policies. There were mixed findings across multiple dimensions which lend themselves to the constantly evolving nature of organizational guidelines in quarantining, masking, social distancing, and spatial considerations among COVID-positive patients (Supplementary file 1).Table S1 presents a comprehensive breakdown of the programs sampled along with their COVID-clearance policies for acute psychiatric admission following a COVID-positive test among children and adolescents.

 A couple programs accept patients with COVID-positive status. However among them, some require patients to self-isolate for their treatments until they have a negative retest while others will allow patients to participate in the breadth of treatment with fellow patients as long as they are masking and social distancing in group contexts on the units. In another program, there was a COVID-designated space on the unit for COVID-positive patients to participate in group therapies. One program had a standalone COVID-designated psychiatric unit for patients and patients could be admitted there as long as there was bed space. In different programs, COVID-positive patients could be admitted as long as there were single rooms open for self-isolation. In programs that required self-isolation, patients either could not participate in groups with fellow patients or could virtually through technology via tablets.

 In addition, number of days for quarantining following a post-COVID-19 positive test result varied substantially among the programs sampled. Some programs required as low as 3 days of quarantine while others involved 5, 6, 7, 10, 11, and up to 14 days of quarantine. Among programs that required negative retests, some of them stipulated one negative retest as a standalone condition for admission while others required one or two negative retests after completing a prescribed number of days for quarantine. For some of the negative retest requirements, a couple programs required two retests that were either 24 or 48 hours apart while others only required one retest 24 hours prior to admission. Additional programs required solely one negative retest after quarantining over the prescribed number of days. Among programs necessitating COVID-19 retests, some of them stipulated a PCR antigen retest while others accepted results from a rapid test but not a home-based testing kit. There were also variations among programs on whether patients could be symptomatic or asymptomatic after one or more negative retests and completing a prescribed number of days for quarantine. One program was open to accepting patients still experiencing symptoms except fevers. Another program required laboratory testing to assess for white cell count and other levels prior to consideration for admission following a COVID-positive test.

 These mixed findings suggest inconsistencies and fluctuations in the organizational guidelines for quarantine and additional infection control practices prescribed by the CDC. Furthermore, these variations in policies directly have implications for clinical practice during a critical time for needed acute crisis stabilization which can certainly be a predictor of treatment outcomes. In addition, it may also take up to 3 months or potentially longer for patients to test negative following an initial COVID-positive test. Among programs that require a negative retest, attaining consideration of admission into these programs does not appear realistic based on this parameter. It follows that the nature of this parameter itself creates inequities in the provision of psychiatric care for these patients since had they not tested positive, then they could have accessed the depth and breadth of services in these programs.

 Several of the admissions departments among child and adolescent acute inpatient psychiatric programs in this sample reported that they followed the CDC guidelines in determining eligibility for admission from a COVID-clearance perspective. Findings from this study also directly tie into the constantly evolving nature of these COVID-19 behavioral guidelines and mixed messaging provided by the government which directly contribute to the inaccessibility, limitations, and fragmentation of mental health care services for patients needing acute crisis stabilization. It follows that these inconsistencies could further exacerbate our existing global mental health crisis.

## Implications for needed psychiatric care

 Based on our findings and our own clinical practice, patients with COVID-19 positive test results who are admitted on the medical units and medically cleared for inpatient psychiatric placement are not able to make the transition given their COVID-19 positive status. In different instances, there are also patients in our healthcare system currently in the midst of receiving treatment on either inpatient psychiatric or eating disorder units who are sent back to the medical units after testing positive for COVID-19. Subsequently, their psychiatric treatment is at a standstill at this point given their COVID-19 positive status.

 This reality further heightens the risk that children may not make progress in meeting their treatment goals. Given their increased vulnerability surrounding this time of crisis, they are significantly more susceptible to regression, relapse, and decompensation from not achieving acute crisis stabilization within a time sensitive duration. In our own practice, we have also seen children with COVID-19 return home with safety planning provided by the consulting child psychiatry team. Of note in these instances, the initial recommendation from psychiatry was inpatient psychiatric placement for these children. In several of these instances, psychiatry has changed their clinical impression for disposition amidst boarding and subsequently recommended safety planning and discharge to home with outpatient follow-up in the community for mental health services. However, there are limitations in this approach. Scheduling an intake for outpatient therapy and psychiatric evaluation will likely not happen instantly. In addition, safety planning can only extend so far with crisis intervention. Oftentimes, a goal of acute crisis stabilization in the inpatient psychiatric context is to create a more comprehensive and realistic safety plan in a psychologically safe and controlled space with children to reduce the risk of future self-harm and psychiatric morbidity. By not having this space, safety planning may not yield the most psychological benefits for the child and in turn heighten the risks of harm than the benefits of safety. Furthermore without this space, there is also increased risk of sending children with unmet psychiatric needs home without the resources that they truly need to navigate this time of crisis, thereby heightening potential inequities in access to psychiatric care for these patients.

## Operational implications

 From an operational perspective, there is a persistent concern that these patients are taking up bed space when otherwise medically stable. Further, this reality raises ethical implications that there are more acutely ill patients who may need these beds and are unable to attain them. From a cost efficiency standpoint, boarding also exerts financial strain on the healthcare system given that it is challenging to make a compelling case for financial coverage of days without any true interventions when patients are not receiving any psychiatric treatment and also do not necessitate any medical interventions. There is clearly more work needed to navigate these challenges in operating a healthcare system that meets the needs of these patients and aligns with our existing population trends in addressing the growing mental health crisis.

## Staffing and architectural considerations

 It is crucial to account for the significant staffing shortages prominent in the healthcare landscape during this time. In fact, this staffing crisis contributes to the scarcity of inpatient psychiatric care for a range of patients inclusive of children testing positive for COVID-19. Time, resources, and critical examination of the existing staffing needs by institutional stakeholders to meet the care needs of these patients is warranted across healthcare systems. In addition, determining the appropriate space and landscape considerations in a biodome psychiatric unit configuration could yield promise in increasing the delivery of acute psychiatric services for patients across healthcare systems. Hospital administration teams could learn more specifically from the units at the University of Rochester Medical Center, the hospital in Israel, and the Bellin Psychiatric Center on factors that worked and did not work in designing these spaces for patients. Developing an acute psychiatric care model that prioritizes patient care to the highest standard for children with unmet mental health needs presenting with crisis and testing positive for COVID-19 could contribute towards the larger goal of the World Health Organization, Sustainable Developmental Goals of the United Nations, and Healthy People 2030 in increasing accessibility, quality, and equity in mental health care for individuals on both global and national levels.

## Considerations for extending coverage of the boarding crisis within the mental health crisis

 Of note, there are multiple campaigns and initiatives on community, national, and global levels that seek to heighten knowledge and awareness on mental health and further also advocate for achieving parity in mental health across a range of determinants (e.g. World Health Organization’s World Mental Health Day, Jansport’s Lighten the Load and Maybelline’s Brave Together campaigns). Boarding amidst COVID-19 positive status, scarcity of resources, and additional factors collectively represents one dimension that is not covered in the aforementioned campaigns and additional ones in this domain. It follows that integrating this content into prominent campaigns centered on considerations in mental health can heighten the visibility of boarding as a challenge contributing to the existing global mental health crisis and in turn could mobilize more advocacy and resources in collaboration across community organizations, foundations, and healthcare systems on both national and global levels. In addition, extending this content into social media could also heighten knowledge and awareness on its implications. Primarily, healthcare systems are well-aware of the boarding crisis within the mental health crisis. However, the rest of the lay population is not well-versed on the intricacies and complexities surrounding this embedded crisis. It follows that enlisting healthcare providers to take a more visible presence across traditional and nontraditional media platforms (e.g. television, magazines, newspapers, and social media) could also heighten knowledge and awareness as the basis to reach more stakeholders around the world that could help in mitigating boarding as a risk factor and in turn contribute towards addressing our global mental health crisis.

## Final thoughts and considerations

 Unfortunately based on our findings and from the existing literature, there is not sufficient information on best practices to navigate this crisis. However, we are now into year three of the pandemic. COVID-19 does not appear to be disappearing anytime soon as this pandemic is transitioning into an endemic and thereby becoming integrated into the mainstream culture of our world. It follows that this current chronic COVID-19 era presents a window of opportunity to further explore how the interface of the pandemic and the growing mental health crisis could be mitigated through increasing access to acute psychiatric services for so many of our patients around the world.

## Competing Interests

 The authors declare that they have no competing interests.

## Data Availability Statement

 Not applicable.

## Ethical Approval

 Not applicable.

## Funding

 Not applicable.

## Supplementary Files

Supplementary file contains Table S1.
